# Monitoring Knowledge, Attitudes, and Practices on Restraint Use in Adult and Pediatric Intensive Care Units: The Multicenter Development and Validation of the CON-Ti-IT Questionnaire

**DOI:** 10.3390/nursrep16010010

**Published:** 2025-12-25

**Authors:** Loredana Dittura, Silvana Schreiber, Valentina Guidi, Manuela Giangreco, Giulia Zamagni, Erica Venier, Raffaella Di Meola, Elisabetta Balestreri, Giorgia Toso, Patrizia Sartorato, Luca Bertocchi, Sara Buchini, Raffaella Dobrina

**Affiliations:** 1 Institute for Maternal and Child Health IRCCS Burlo Garofolo, Via dell’Istria, 65, 34137 Trieste, Italy; loredana.dittura@burlo.trieste.it (L.D.); silvana.schreiber@burlo.trieste.it (S.S.); manuela.giangreco@burlo.trieste.it (M.G.); giulia.zamagni@burlo.trieste.it (G.Z.); sara.buchini@burlo.trieste.it (S.B.); raffaella.dobrina@burlo.trieste.it (R.D.); 2 Azienda Sanitaria Universitaria Giuliano Isontina, Via Giovanni Sai, 2/71, 34128 Trieste, Italy; valentina.guidi@asugi.sanita.fvg.it (V.G.); erica.venier@asugi.sanita.fvg.it (E.V.); raffaella.dimeola@asugi.sanita.fvg.it (R.D.M.); elisabetta.balestreri@asugi.sanita.fvg.it (E.B.); giorgia.toso@asugi.sanita.fvg.it (G.T.); patrizia.sartorato@asugi.sanita.fvg.it (P.S.); 3 William F. Connell School of Nursing, Boston College, Chestnut Hill, Boston, MA 02467, USA

**Keywords:** restraint, knowledge, attitude and practice, nursing, intensive care unit, pediatric intensive care unit, restraint assessment, restraint monitoring

## Abstract

**Background/Objectives:** The use of physical restraints in adult and pediatric intensive care units (ICUs) is common yet controversial. While restraints are intended to prevent treatment interference or self-harm, they pose significant physical, psychological, and ethical risks. Nurses in intensive care units play a key role in decisions about restraint application, but there is a global lack of validated tools to assess their knowledge, attitudes, and practices, particularly in non-English-speaking contexts. Aim of this study was to develop and validate a questionnaire for assessing knowledge, attitudes, and practices (KAP) of ICU nurses regarding restraint use in adult and pediatric settings. **Materials and Methods:** A multi-method psychometric validation study was conducted across both adult and pediatric ICU settings at two hospitals in northern Italy. Questionnaire development included literature review, expert consultation, and iterative content and face validity assessments. Reliability was tested using test–retest methods, and construct validity was explored through exploratory factor analysis. The study followed COSMIN guidelines. **Results:** The final CON-Ti-IT questionnaire comprised 29 items across three subscales: Practices, Attitudes, and Knowledge. It demonstrated strong content validity (CVI = 0.96) and good internal consistency for the Practices subscale (Cronbach’s α = 0.89). Internal consistency for the Attitudes (α = 0.51) and Knowledge (α = 0.47) subscales was lower, reflecting the broader conceptual variability of these domains. Exploratory factor analysis confirmed the structural validity of the tool and led to the removal of three items with low factor loadings. **Conclusions:** This study presents the first validated tool specifically designed to evaluate ICU nurses’ KAP on restraint in adult and pediatric settings. While developed and validated in Italy, it could undergo cross-cultural adaptation and translation for use in other languages and healthcare systems. Its strong psychometric properties support its application in future research, and the data collected through its use can serve both to improve patient care and to provide a foundation for targeted educational initiatives.

## 1. Introduction

Worldwide, 23–75% of patients in adult intensive care units (ICU) are restrained [1]. In pediatric ICUs, prevalence data are more limited [2]. A multicenter study in 26 Japanese pediatric ICUs reported restraint use in 53% of children, with handcuffs most common [3]. Restraints are employed for safety purposes, primarily to prevent patients from harming themselves or inadvertently removing essential medical devices during episodes of agitation (e.g., endotracheal tubes, arterial or central intravenous lines) [4,5,6,7]. In pediatric ICUs, restraint use is especially associated with patients experiencing anxiety, insufficient analgesia or sedation, pharmacological withdrawal symptoms, or high risk of accidental device removal or falls [8].

However, studies show they may have unintended effects, such as increasing the risk of self-extubation [9], worsening agitation, and causing physical and psychological harm or even death [6,10,11,12]. Restraints also impose a significant emotional burden on family caregivers [13] and raise serious ethical concerns, particularly in pediatrics, where the right to physical and psychological protection is paramount [2,14,15].

Despite these risks, restraints are frequently applied before exploring less invasive alternatives such as symptom monitoring, reduced device dwell time, distraction techniques, or family presence, especially in children [2,16]. Although the decision to use restraints is inherently multidisciplinary, nurses play a central role, as their knowledge and attitudes strongly influence practice and can shape the overall decision-making process [5,7,17]. However, not all nurses are acquainted with the options of alternatives to restraints, know how to make more informed decisions about their use or experience moral distress when restraining a patient, which can lead to restraints being applied more readily [4,5,16,18,19].

These findings highlight the urgent need to apply guidelines in all adult and pediatric ICU settings, which include developing tailored educational programs to minimize restraint use and monitoring healthcare providers’ knowledge, attitudes and practices (KAP) of restraint [4,15,20,21]. A brief conceptual clarification of the KAP framework is warranted. In this model, knowledge refers to nurses’ understanding of indications, risks and alternatives to restraint; attitudes reflect their beliefs and ethical positions; and practices denote their actual clinical behaviors. Recent nursing literature shows how these components interact in shaping restraint-related decisions [22].

Despite the global importance of reducing restraint use in healthcare, and particularly in ICUs, few psychometrically validated questionnaires exist, especially ones adapted to ICU-specific contexts and cultural settings. Existing tools in Chinese [23], Malay [24], and Turkish [25] show variable internal consistency (Cronbach’s α from 0.59 to 0.947). A recently validated Spanish questionnaire for pediatric ICUs reported good reliability, with α = 0.84 for attitude and 0.75 for intention subscales [8]. These findings highlight the need for context-specific tools to capture local cultural, ethical, and professional nuances. Such tools are essential for accurate data collection, meaningful international comparisons, and targeted intervention planning. The current study was conducted in Italy, where cultural, legal, and ethical norms regarding restraint use may differ from those in other countries. In the Italian Friuli Venezia Giulia Region, legislation supports restraint-free environments, prioritizing patient safety and dignity [26]. A Commission was established to assess restraint use in all healthcare settings and educational programs have been provided. Despite these efforts, the prevalence of restraint use remains underreported [27] and, to our knowledge, no validated tools are available in Italian language to address this gap, particularly because existing instruments are not ICU-specific, lack validation in the Italian cultural context, and do not incorporate both adult and pediatric care aspects, with most originating from other clinical settings such as mental health or gerontology [28].

### Aims and Research Questions

This study aimed to develop and validate a questionnaire to assess ICU nurses’ KAP regarding restraints in both adult and pediatric settings. The research activities in this study were guided by the following research questions:

(1) What key factors should be monitored in adult and pediatric ICUs to identify and control nurses’ restraints’ use?
(2) Can a single questionnaire effectively assess nurses’ KAP on restraints in both adult and pediatric ICUs?
(3) What are the most relevant items for a questionnaire assessing nurses’ KAP on restraints in adult and pediatric ICUs?
(4) What is the most appropriate response format for accurately assessing nurses’ KAP on restraints?

## 2. Materials and Methods

A multi-method, multicenter questionnaire validation study was conducted from 2020 to 2023, following the framework of Rattray and Jones [29]. It included a 10-bed maternal and child ICU and recovery room (Center A) and adult ICUs, cardiology ICUs, and recovery rooms across three towns in northeastern Italy (Center B), totaling 91 beds. The reporting of the process was checked against relevant items of the COnsensus based Standards for the selection of health status Measurement INstruments (COSMIN) guidelines [30].

### 2.1. The Questionnaire Development and Validation Process

**Figure 1:**
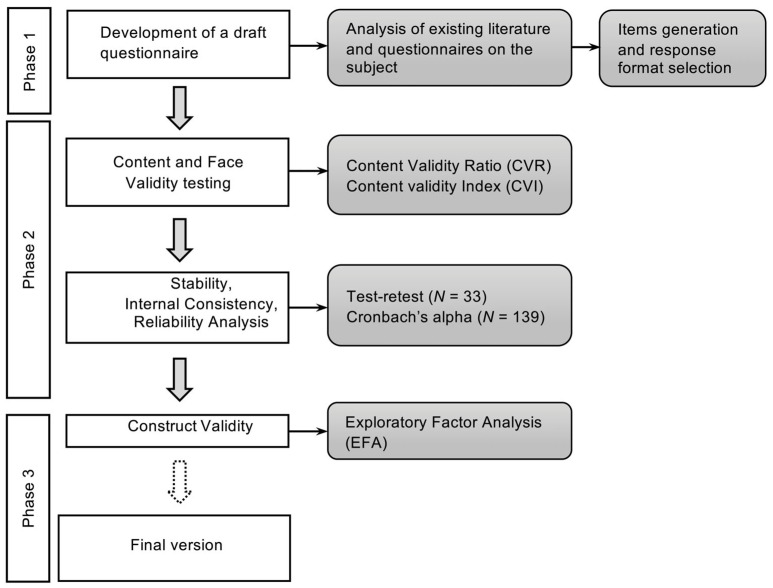
The development and validation process of the CON-TI-iT questionnaire.

The development and validation process of the CON-Ti-IT questionnaire involved three phases (Figure 1):

(1) Development of a draft questionnaire.
(2) Psychometric testing of the questionnaire.
(3) Construct validity.

#### 2.1.1. Phase 1—Development of a Draft Questionnaire

A panel of experts from the two Centers, including adult and pediatric ICU nurses, head nurses, and nurse managers, contributed to the instrument development. Their sociodemographic and professional characteristics are reported in Table 1. under the column “Content validity.” The panel met periodically to review the research questions and evaluate the items following the KAP framework [31], using a structured content-validity procedure. A researcher (RD) facilitated the process, and all members were kept updated throughout the development phases. A structured content-validity procedure was used.

A literature review was performed by three experts (LD, SB, RD) to identify examples of questionnaires designed to assess nurses’ KAP of restraints in ICUs or other settings. Retrieved studies to be considered eligible in this literature review had to: (1) be published from 2010 to 2020; (2) be published in English or Italian; (3) be freely available in full-text from the research online library of Center A. The main search string used was: “knowledge” or “attitude” or “practice” or “KAP” and “restrain*” and “hospital” or “intensive care unit” or “ICU” and “nurse” and “questionnaire” and “validat*”. After three months, the researchers reported their findings to the group by describing the questionnaires they identified in terms of structure, items and different response formats. All experts had a good understanding of written English so a hard copy of the questionnaires was distributed for review. Findings from the review were discussed to support decisions on the type and appropriate number and order of items; the best response format and options for open-ended, free text responses. A first draft of the questionnaire was developed at this stage.

#### 2.1.2. Phase 2—Psychometric Testing of the Questionnaire

##### Content and Face Validity

The first draft of the questionnaire was evaluated for content and face validity. For content validity, the panel of experts was asked to classify each item as “essential,” “not essential,” or “uncertain,” in accordance with Lawshe’s method [32,33]. Based on their responses, the Content Validity Ratio (CVR) was calculated for each item. Items that did not reach the minimum threshold of 0.78 were revised or removed. Clarity was quantitatively assessed using the Item-level Content Validity Index (I-CVI) as described by Polit et al. [34]. Experts rated the clarity of each item on a 4-point scale (1 = not clear, 4 = very clear). The I-CVI for clarity was calculated as the proportion of experts rating an item as either 3 or 4, and the Scale-level CVI/Average (S-CVI/Ave) was computed as the average of the I-CVI values across all items. Items with I-CVI < 0.78 were revised and re-evaluated until they met the threshold.

##### Reliability Analysis

Test–retest analysis was performed to confirm the questionnaire’s reliability and stability. The questionnaire was administered at baseline (T0) to eligible nurses in Centers A and B through convenience sampling. The head nurses of the wards involved distributed the hard copy of the questionnaire along with a cover letter and a demographic and professional form. After 10 days (T1), the questionnaire was administered again to the same respondents. This timeframe, falling within the generally recommended range of 1–2 weeks, helps minimize recall bias while maintaining stability in participants’ conditions [35]. A code was assigned to each participant at baseline to pseudo-anonymize the response sheets and to be able to match (T0) and (T1) questionnaires for data analysis. The completed questionnaires were collected in a box present in the head nurses’ office in the ICU wards. Time for completion was registered.

The strength of agreement was categorized as follows: “poor” or “absent” for kappa values of 0.2 or less, “fair” for kappa values between 0.21 and 0.4, “moderate” for kappa values between 0.41 and 0.60, “good” for kappa values between 0.61 and 0.80, and “very good” for kappa values greater than 0.80 [36]. The questionnaire was subsequently tested on a larger convenience sample of nurses for final validation and completed online via Research Electronic Data Capture platform (REDCap 14.0.17—© 2024 Vanderbilt University), along with a demographic and professional form.

##### Phase 3. Construct Validity

Exploratory Factor Analysis (EFA) was performed to inspect the construct validity of the questionnaire and to provide preliminary evidence on the underlying factor structure. First, Kaise-Meyer-Olmin (KMO) and Bartlett’s sphericity tests were conducted to evaluate sampling adequacy and factorability. The number of factors to retain was determined using the elbow method, which identifies the point in the scree plot where the decrease in eigenvalues levels off (i.e., the ‘elbow’), indicating that additional factors provide minimal explanatory value. After determining the factor structure, only items with factor loadings greater than 0.30 were retained, ensuring that each item was meaningfully associated with its corresponding factor, as recommended for preliminary exploratory analyses [37,38]. To enhance the interpretability of the factor structure, Varimax rotation was applied. Varimax is an orthogonal rotation method that maximizes the variance of squared loadings of each factor, resulting in a factor solution where each factor is clearly defined by a high loading on a few items and low loadings on others. This rotation facilitates a clearer and more meaningful interpretation of the factors.

### 2.2. Participants

The nine members of the experts were involved in the content and face validity of the questionnaire. A convenience sampling was adopted to enroll nurses for the reliability analysis. Nurses were invited to participate through nurse coordinators and they were included in the study if they worked in adult or pediatric ICU of Center A or B. Nurses working in neonatal ICU were excluded. Agency staff, temporary personnel, and newly hired nurses (<3 months) were not eligible. Nurse managers or educators were excluded from the psychometric validation phase to ensure that responses reflected bedside clinical practice. Recruitment occurred through unit nurse coordinators, who distributed the study information sheet and either paper questionnaires (first phase) or REDCap survey links (subsequent phases) sent through institutional e-mails. Participation was voluntary and anonymous. Response rates were monitored based on the number of questionnaires returned relative to those distributed in each ICU. Representation of adult and pediatric ICU nurses was ensured by inviting all eligible nurses from both settings. Sample size was calculated for reliability testing, in particular for test–retest and Cronbach alpha calculations using the questionnaire by Cui et al. [23] as an example.

(1) Test–retest: in a test for agreement between two test administrations using the Kappa statistic, a sample size of 33 nurses achieves 80% power to detect a true Kappa value of 0.85 in a test of H0: Kappa = 0.50 vs. H1: Kappa ≠ 0.50 when there are five categories with frequencies equal to 0.50, 0.30, 0.10, 0.05, and 0.05. This power calculation is based on a significance level of 0.05.
(2) Cronbach alpha: a sample of 130 nurses was estimated assuming an internal consistency in which in the null hypothesis, the Cronbach alpha is equal to 0.55, and in the alternative hypothesis, Cronbach’s alpha is at least equal to 0.70. Power is set at 80%, the value of alpha at 0.05 and the number of items equal to 4.

### 2.3. Ethical Considerations

The study was approved by the Institutional Review Board of Center A (IRB-BURLO 02/2020 20 April 2020). Prior to each validation phase, participants received an information sheet explaining the study and signed an informed consent form. Anonymity and confidentiality were ensured; paper questionnaires were stored in a locked cabinet in the researchers’ office, accessible only to the study team, and no incentives or compensation were provided. Due to the ICU nurses’ workload during the COVID-19 outbreak [39], researchers opted not to involve them at that time. Consequently, an amendment extending the study period was approved by the Regional Ethics Committee (IRB-BURLO 01/2021 14 January 2021). Following this amendment, data collection shifted to the REDCap online platform, which ensured secure, password-protected data management and prevented potential COVID-19 transmission via contaminated surfaces [39].

### 2.4. Data Analysis

Categorical variables were presented as counts and percentages. For continuous variables, which deviated from normal distribution as indicated by the Shapiro–Wilk test, the median and interquartile range (IQR) were reported. The Chi-square goodness-of-fit test was used to evaluate the distribution of categorical variables. Statistical significance was defined as α = 0.05. Data analysis was performed using StataCorp. 2023. Stata Statistical Software: Release 18. College Station, TX, USA: StataCorp LLC.

## 3. Results

The instrument was evaluated across three sequential phases involving a total of 181 participants: 9 experts for content validity, 33 nurses for the pilot testing, and 139 nurses for the main validation sample. Their sociodemographic and professional characteristics are summarized in Table 1.

### 3.1. Phase 1: Development of a Draft Questionnaire by the Panel of Experts

- Analysis of existing literature and questionnaires on the subject

Three experts (LD, SB, RD) conducted a literature review of studies published from 2010 to 2020 in English or Italian, freely available in full-text from the research online library of Center A. The main search string used was: “knowledge” or “attitude” or “practice” or “KAP” and “restrain*” and “hospital” or “intensive care unit” or “ICU” and “nurse” and “questionnaire” and “validat*”. Then, after about three months, the researchers reported their findings to the group by describing the questionnaires they identified in terms of structure, items and different response formats. Some of the questionnaires presented were specific to the ICU setting [20,23,28,40], while others were from general medical-surgical wards or nursing homes [2,41]. All experts had a good understanding of written English so a hard copy of the questionnaires was distributed for review. The experts agreed to develop a new questionnaire rather than merely adapting and validating an existing one in Italian. This decision was based on several factors: some available tools contained an excessive number of items (e.g., 40 or 72), which experts considered overly burdensome for nurses to complete [20,23,28]; other tools did not account for certain forms of restraints, such as chemical restraints [23]; some questionnaires included only a few items addressing the challenges of teamwork in restraint management [28]. Table 1 includes all the socio-demographic and professional characteristics of the participants across the various phases of the questionnaire validation.

b. Items construction and scale selection

Using the examples of the questionnaires retrieved from literature and focusing on the three dimensions of restraint that the study group wanted to explore with the questionnaire (e.g., nurses’ KAP) the experts began constructing its structure and items. The development process took almost a year, with the instrument undergoing multiple revisions during meetings and e-mail exchanges with experts. Items were built and then removed, changed or rewritten a lot of times. The first draft of the questionnaire consisted of 37 items with responses 5-point Likert scale ranging from 1 = “Rarely” to 5 = “Usually” along with four open-ended questions. For example, items exploring monitoring times of restrained patients monitoring or open-ended questions exploring knowledge of Italian legislation were eliminated. The final version, before validity analysis, consisted of 32 items with responses on a Likert scale from 1 = “never” to 5 = “always” for the “practice” and “attitude” subscale, and from 1 = “I totally disagree” to 5 = “I totally agree” for the “knowledge” subscale. A name was discussed for the questionnaire and the final agreement was: (Italian version): “Conoscenze, atteggiamenti e pratiche sulla contenzione degli infermieri nelle unità di terapia intensiva pediatrica e per adulti” with acronym: “CON-Ti-IT” (in English: “Knowledge, attitudes, and practice of pediatric and adult ICU nurses regarding restraints”).

### 3.2. Phase 2: Psychometric Testing of the Instrument

#### 3.2.1. Content and Face Validity

Content validity analysis confirmed that all items met the minimum thresholds for both relevance (CVR ≥ 0.78) and clarity (I-CVI ≥ 0.78). The S-CVI/Ave for clarity was 0.92, with I-CVI clarity values ranging from 0.78 to 1.00. For example, when 8 out of 9 experts rated an item at the highest clarity levels (scores of 3 or 4). Feedback from the nine experts led to targeted adjustments in terminology and phrasing to improve comprehension and alignment with adult and pediatric ICU specific clinical language. Following these revisions, the final pre-validation version of the questionnaire consisted of 32 items.

#### 3.2.2. Reliability

For test–retest 33 nurses were enrolled with data collection starting in October 2020. The strength of agreement between items responses in T0 and T1 was “good” to “very good” (test–retest results are presented in Supplementary File S1. The reported time for completion of the questionnaire was below ten minutes for all nurses. The sample size required for reliability testing was achieved, with a total of 139 nurses enrolled. The internal consistency of the questionnaire was good, as indicated by a Cronbach’s alpha of 0.86. Detailed results are presented in Table 2. The subscales exhibited varying levels of internal consistency: the “Practices” subscale showed a Cronbach’s alpha of 0.89, while the “Attitudes” and “Knowledge” subscales showed alphas of 0.51 and 0.41, respectively. Lower internal consistency values were theoretically anticipated and considered acceptable for the two latter domains. For this reason, the Attitudes and Knowledge subscales were retained despite their lower alpha coefficients, in line with the conceptual heterogeneity intentionally embedded in these dimensions. In the Attitudes subscale, item–total correlations ranged from 0.13 (item 20) to 0.45 (item 19), while in the Knowledge subscale they ranged from 0.07 (item 27) to 0.51 (item 29), further supporting the expected variability across items.

### 3.3. Phase 3. Construct Validity

The KMO value of 0.77 indicated good sampling adequacy. In addition, Bartlett’s test was significant (χ^2^ = 1963.83, *p* < 0.001), confirming that the correlations among items were sufficient to proceed with factor analysis.

Factorial analysis yielded four factors in “Practices” subscale, two in “Attitudes” subscale and three in the “Knowledge” subscale (Table 2). Three items in the “Knowledge” subscale had loadings below 0.30 and were removed from the final questionnaire. The final structure explained 47% of the variance for the “Practices” subscale, 29% for “Attitudes,” and 26% for “Knowledge”.

The final, validated questionnaire consists of 29 items divided into three subscales, namely 17 items in the “Practices” subscale, six items in the “Attitudes” subscale and six in the “Knowledge” subscale (Supplementary Files S2 and S3).

## 4. Discussion

Monitoring the phenomenon of restraints to identify malpractices and educational needs and providing appropriate feedback to staff are some of the interventions suggested by the literature to prevent its use in adult and pediatric settings [15,21]. However, it is essential that the questionnaires used in healthcare, including those for monitoring the use of restraints, are validated for reliability and validity [29].

In summary, our findings indicate that the CON-Ti-IT questionnaire demonstrated strong content and face validity, acceptable reliability, and a clear factor structure that supports its use for assessing ICU nurses’ knowledge, attitudes, and practices regarding restraint. To the best of our knowledge, this study represents the first development and validation process of a questionnaire specifically designed to assess ICU nurses’ KAP regarding the use of restraints for use in both adult and pediatric settings. The results demonstrated high reliability and strong construct and face validity, effectively addressing a significant gap in the existing literature. Beyond statistical relevance, the face validity assessment strengthened the instrument’s clinical usability by ensuring the items were understandable and contextually appropriate for both adult and pediatric ICU nurses. This alignment between conceptual content and practical clarity is essential in enhancing user engagement and real-world application [29]. While adult and pediatric ICU settings differ, a unified instrument is justified by the clinical reality in hospitals, especially maternal and child health centers as the one where this study was conducted, where the ICU admits both pediatric patients (including near-adults) and adult women with obstetric complications.

Content validity analysis confirmed that the final items aligned well with the study’s aims [29]. This ensures the instrument accurately captures the essential aspects of KAP in the context of ICU restraints. The reliability analysis also demonstrated that the questionnaire, particularly the “Practices” subscale, consistently measures this dimension. A closer examination of the psychometric results shows that the behavior of the CON-Ti-IT subscales is consistent with what has been observed in instruments assessing multidimensional constructs. Attitudes and declarative knowledge often demonstrate lower internal consistency because they encompass heterogeneous experiential, ethical, and contextual components rather than a single unified latent trait. This pattern has been documented in previous nursing studies, including those examining restraint-related constructs and other complex behavioral domains [17,20,28]. The lower internal consistency observed in the “Attitudes” and “Knowledge” subscales therefore reflects the broader conceptual diversity inherent to these domains.

Only three items were deleted during factor analysis. Interestingly, two of these items were related to agitated or confused patients suggesting that these concepts may be more complex or context-dependent than originally thought [42]. Their removal improved the overall construct validity of the questionnaire and allowed for a more focused and interpretable factor structure. The content aligns with other questionnaires on the subject, covering aspects such as the conditions for using restraints in ICUs, the involvement of family, and nurses’ attitudes toward their application [8]. In particular, three items concerned the role of family that was considered crucial in a pediatric setting but increasingly recognized as important in adult ICUs as well [7]. This alignment with other tools in the field enhances the relevance and applicability of the questionnaire, making it a valuable instrument for monitoring and improving the practices surrounding the use of restraints in both adult and pediatric ICUs. However, unlike other available questionnaires [20,23,28], the limited number of items in this questionnaire facilitates quick completion, minimizing respondent burden while ensuring comprehensiveness. Moreover, the questionnaire includes an item investigating the practice of chemical restraint (e.g., sedatives), an aspect often overlooked in similar assessments. While the clinical use of sedatives is common in ICUs, their application without clearly defined goals for therapeutic sedation raises ethical and regulatory concerns [43] and highlights the need for clear nursing standards and accountability. Recent literature has defined chemical restraint as the intentional use of sedatives to control behavior or prevent physical restraint, emphasizing structured, ethical nursing practices to guide its use [44]. Furthermore, four items (e.g., n. 15–17 and 20) address the challenges of teamwork in restraint management, a crucial factor influencing adherence to best practices and ethical considerations [16].

A key strength of this study is its multi-center design, being conducted in both adult and pediatric ICU settings. This diversity enhances the generalizability of the findings [45] and makes the questionnaire applicable in a range of ICU environments. Moreover, the detailed development process, including peer-review by experts of two hospitals, the iterative revision, and psychometric testing, ensures that the final questionnaire is both methodologically and practically applicable.

Moreover, the present findings highlight the importance of a validated tool tailored to the Italian context, given the cultural and regulatory variability in restraint practices worldwide. While some Italian regions, such as Friuli Venezia Giulia, promote restraint-free care, national standards remain inconsistent. Italian pediatric care prioritizes relational approaches and family involvement, as highlighted in the Charter of Children’s Rights in Hospital [14]. Internationally, restraint practices differ markedly. In China and Malaysia, decisions are often protocol-based and shaped by institutional risk norms [20,23,24], whereas United Kingdom nurses express ethical discomfort and seek clearer policies [7]. Recent Spanish findings show that restraint intentions vary with nurses’ sociodemographic factors and training exposure [8]. The CON-Ti-IT tool responds to this variability by assessing not only standard practices, but also contextual factors such as staff shortages, ethical tensions among colleagues, and fallback strategies like improvised restraints. It also captures knowledge and attitudes related to team decision-making, legal awareness, and family inclusion, aligning with Italy’s relational care ethos. Such a context-sensitive instrument is essential for guiding education, auditing, and policy reforms tailored to Italian ICUs, while enabling meaningful comparison with international data.

This study presents a few methodological limitations. Given the sensitive nature of restraint use, responses may have been influenced by social desirability bias, despite the anonymity of data collection. Moreover, participants were selected through a convenience sampling method, which, although practical for preliminary validation purposes, may have resulted in a sample that is not fully representative of the broader nursing population, potentially limiting the generalizability of the findings. In addition, confirmatory factor analysis was not performed due to sample size limitations. A future study using an independent sample is needed to confirm the stability of the proposed factor structure.

Although experts involved in item development represented both adult and pediatric ICUs, participant distribution during psychometric testing was unbalanced, potentially affecting the representation of pediatric-specific perspectives. Finally, criterion validity could not be assessed, as KAP scores were not compared with observed restraint practices or with outcomes from restraint-related training. Future studies should examine these associations to further strengthen the instrument’s validity.

### Relevance to Clinical Practice

Regular assessment with validated tools like CON-Ti-IT can help identify specific educational needs, support clinical audits by highlighting gaps in current practice, and inform quality improvement efforts. The CON-Ti-IT questionnaire offers a concise, reliable means to assess these factors within adult and pediatric ICU settings and can provide aggregated data that inform organizational decision-making and the development of restraint-reduction policies. To effectively reduce restraint use, such assessments should be integrated into comprehensive restraint reduction programs that include targeted education, systemic interventions, and multidisciplinary collaboration, thereby promoting ethical, patient-centered care and enhancing patient safety and dignity.

## 5. Conclusions

In this study, we developed and validated the 29-item CON-Ti-IT questionnaire to assess ICU nurses’ knowledge, attitudes, and practices regarding the use of restraints in adult and pediatric intensive care settings. The instrument demonstrated good content validity, an interpretable factor structure corresponding to the three intended domains, and acceptable reliability, with strong test–retest stability and high internal consistency for the Practices subscale. These findings indicate that the CON-Ti-IT questionnaire is a valid and reliable tool for evaluating KAP related to restraint use in ICU settings. Further research is needed to strengthen the questionnaire’s psychometric properties and broaden its applicability. This includes exploring cross-cultural adaptation and translation into other languages and healthcare systems. To verify the instrument’s factor structure, CFA should be conducted on larger and more diverse samples [29]. Longitudinal studies may also help assess how KAP scores relate to actual restraint practices and patient outcomes over time. Finally, while developed and validated in Italy, the questionnaire could undergo cross-cultural adaptation and translation for use in other languages and healthcare systems.

## Figures and Tables

**Table 1:** Socio-demographic and professional characteristics of the participants across the various phases of the questionnaire validation process.

Variables	Content Validity	Test–Retest	Reliability
Sample size (*n*)	9	33	139
Sex, *n* (%)			
Male	0 (0)	11 (33)	36 (25.9)
Female	9 (100)	22 (7)	103 (74.1)
Age groups, *n* (%)			
≤25 years	—	—	15 (10.8)
26–30 years	—	10 (30)	27 (19.4)
31–35 years	—	7 (21)	37 (26.6)
36–40 years	1 (11.1)	3 (9)	8 (5.8)
41–45 years	2 (22.2)	4 (12)	14 (10.1)
46–50 years	1 (11.1)	5 (15)	14 (10.1)
51–55 years	4 (44.5)	3 (9)	13 (9.4)
≥ 56 years	1 (11.1)	1 (3)	11 (7.8)
Profession, *n* (%)			
Nurse	4 (44.4)	33 (100)	137 (98.6)
Pediatric nurse	5 (55.6)	0 (0)	1 (0.7)
Not declared	—		1 (0.7)
Units, *n* (%)			
ICU 1 (13 beds)	—	17 (52)	19 (13.7)
ICU 2 (6 beds)	—	9 (27)	27 (19.4)
ICU 3 (8 beds)	—	2 (6)	10 (7.2)
CIC 1 (10 beds)	—	3 (9)	3 (2.2)
CIC 2 (10 beds)	—	1 (3)	38 (27.3)
CIC 3 (8 beds)	—	1 (3)	3 (2.2)
RR 1 (20 beds)	—	—	10 (7.2)
RR 2 (16 beds)	—	—	28 (20.1)
Not declared	—	1	1 (0.7)
Context, *n* (%)			
Clinical	2 (22.2)		138 (99.3)
Management	7 (77.8)		—
Not declared	—		1 (0.7)
Year since basic diploma/degree, *n* (%)			
≤ 5 years	—	5 (15)	30 (21.6)
6–10 years	—	13 (39)	42 (30.2)
11–15 years	1 (11.1)	1 (3)	19 (13.7)
16–20 years	1 (11.1)	4 (12)	9 (6.5)
≥ 21 years	7 (77.7)	10 (30)	38 (27.3)
Not declared	—	—	1 (0.7)

Legend: CIC, Cardiology intensive care; ICU, Intensive care unit; RR, recovery room.

**Table 2:** Exploratory Factor Analysis (EFA) of the CON-TI-IT questionnaire items.

Item		Factor	F1	F2	F3	F4
Scale 1—Practices	In my work in intensive care unit, I happen to:					
	1#—restrain a patient as they wake up from general anesthesia	Standard clinical practices	\			
	2#—restrain a patient when they are in an altered mental state	Standard clinical practices	0.77			
	3#—restrain a patient when in a state of psychomotor agitation	Standard clinical practices	0.72			
	6#—restrain a patient to prevent the removal of a life-saving device (e.g., endotracheal tube)	Standard clinical practices	0.80			
	7#—restrain a patient to prevent the removal of devices (e.g., nasogastric tube, bladder catheter)	Standard clinical practices	0.81			
	11#—restrain a patient when the team deems it necessary	Standard clinical practices	0.71			
	12#—restrain a patient using wrist restraints	Standard clinical practices	0.67			
	14#—restrain a patient using pharmacological sedation	Standard clinical practices	0.45			
	17#—maintain restraint on a patient when it has been applied by another colleague	Standard clinical practices	0.54			
	8#—restrain a patient when constant observation cannot be guaranteed (e.g., during meetings/handovers)	Clinical practices in difficult contexts		0.69		
	9#—restrain a patient when the workload in intensive care is high (e.g., ongoing emergency)	Clinical practices in difficult contexts		0.79		
	10#—restrain a patient in case of inadequate nurse/patient ratio for the complexity (e.g., lack of staff)	Clinical practices in difficult contexts		0.76		
	13#—restrain a patient through the use of DIY devices (sheets/bandages/bandages)	Clinical practices in difficult contexts		0.40		
	4#—restrain a patient to prevent a fall from the bed	Fall Prevention			0.64	
	5#—restrain a patient to prevent a fall from the chair or armchair	Fall Prevention			0.75	
	15#—face resistance from colleagues when I want to remove the restraints applied to a patient	Sharing with colleagues				0.71
	16#—find myself in disagreement with colleagues and other staff members regarding restraint	Sharing with colleagues				0.70
	***Cronbach’s alpha for the factor***		***0.91***	***0.79***	***0.81***	***0.75***
	***Average Cronbach’s alpha***	***0.89***				
Scale 2- Attitudes	18#—I would feel uncomfortable applying restraints to a patient even if it is to ensure their safety	Attitude towards restraint	0.59			
	19#—In intensive care it is not possible to completely avoid restraint	Attitude towards restraint	−0.42			
	22#—Nurses working in intensive care should never apply restraints	Attitude towards restraint	0.67			
	23#—The patient’s family does not have the right to oppose restraint when it is applied to ensure the patient’s safety	Attitude towards restraint	−0.31			
	20#—The decision to restrain a patient should be shared with the rest of the team	Sharing the decision		0.58		
	21#—The patient’s family must be informed of the reasons that led to the restraint	Sharing the decision		0.57		
	***Cronbach’s alpha for the factor***		***0.60***	***0.60***		
	***Average Cronbach’s alpha***	***0.51***				
Scale 3—Knowledge	29#—Restraint can cause serious complications	Serious consequences of restraint	0.68			
	30#—Restraint can be a cause of death	Serious consequences of restraint	0.70			
	24#—The family can be trained to stay close to the patient in order to avoid restraint in intensive care	Educational aspects		0.44		
	27#—If restraint is applied, it must be documented in the chart indicating the reason, the start and end time and the method with the body area involved at each change in service	Educational aspects		−0.44		
	28#—In intensive care, restraint ensures patient safety	Patient safety			0.31	
	31#—In a confused and agitated patient there are no valid alternatives to restraint	Patient safety			0.32	
	26#—Restraint can be applied when the patient is confused or agitated	nd	nd	nd	nd	nd
	25#—Restraint in intensive care may be applied when constant observation of an agitated patient cannot be guaranteed	nd	nd	nd	nd	nd
	32#—The nurse who applies restraint can be prosecuted by law	nd	nd	nd	nd	nd
	***Cronbach’s alpha for the factor***		***0.73***	***0.45***	***0.30***	
	***Average Cronbach’s alpha***	***0.47***				
	***Average Cronbach’s alpha of the questionnaire***	***0.86***				

Legend: DIY, Do it yourself; F, Factor; nd, not determined.

## Data Availability

The original contributions presented in this study are included in the article/Supplementary Materials. Further inquiries can be directed to the corresponding author.

## Supplementary Materials

The following supporting information can be downloaded at: https://www.mdpi.com/article/10.3390/nursrep16010010/s1, S1: Test–retest analysis; S2: CON-Ti-IT (English version); S3: CON-Ti-IT (Italian version).

## Public Involvement Statement

No public involvement in any aspect of this research.

## Guidelines and Standards Statement

This manuscript was drafted against the COnsensus based Standards for the selection of health status Measurement INstruments (COSMIN) guidelines [30].
