# Supplementary material for: Monitoring Knowledge, Attitudes, and Practices on Restraint Use in Adult and Pediatric Intensive Care Units: The Multicenter Development and Validation of the CON-Ti-IT Questionnaire
Source: Nurs Rep. 2025 Dec 25;16(1):10. doi: 10.3390/nursrep16010010 (PMC12844329; doi:10.3390/nursrep16010010)
Supplement: Supplementary file 1 [file nursrep-16-00010-s001.zip › Supplementary File_S1.pdf]

Supplementary File S1. Test-retest analysis (questions 1-17: value from "never" to "always"; questions 18-31; value from "I totally agree" to "I don't agree").

Item (Italian)	Item (English)	Kappa	P-value	Strength of agreement*
(1-17) Nel mio lavoro in terapia intensiva mi capita di contenere un paziente:	(1-17) In my work in intensive care, I have to restrain a patient:			
1# - Al suo risveglio da un'anestesia totale	1# - As they wake up from general anesthesia	0.74	<.0001	good
2# - Quando è in uno stato confusionale	2# - When they are in an altered mental state	0.77	<.0001	good
3# - Quando è in uno stato di agitazione psicomotoria	3# - When in a state of psychomotor agitation	0.80	<.0001	good
4# - Per prevenire una caduta dal letto	4# - To prevent a fall from the bed	0.79	<.0001	good
5# - Per prevenire una caduta dalla sedia o dalla poltrona	5# - To prevent a fall from the chair or armchair	0.71	<.0001	good
6# - Per evitare che si rimuova un device salvavita (es. tubo endotracheale)	6# - To prevent the removal of a life-saving device (e.g. endotracheal tube)	0.83	<.0001	very good
7# - Per evitare che si rimuova device (es. sondino naso gastrico, catetere vescicale)	7# - To prevent the removal of devices (e.g. nasogastric tube, bladder catheter)	0.83	<.0001	very good
8# - Quando non è possibile garantire un osservazione costante (es. durante riunioni/consegne)	8# - When constant observation cannot be guaranteed (e.g. during meetings/handovers)	0.87	<.0001	very good
9# - Quando il carico di lavoro in terapia intensiva risulta elevato (es. emergenza in corso)	9# - When the workload in intensive care is high (e.g. ongoing emergency)	0.73	<.0001	good
10# - In caso di rapporto numerico infermieri/pazienti inadeguato alla complessità (es. mancanza di personale)	10# - In case of inadequate nurse/patient ratio for the complexity (e.g. lack of staff)	0.69	<.0001	good
11# - Quando l'equipe lo ritiene necessario	11# - When the team deems it necessary	0.89	<.0001	very good
12# - Attraverso l'uso di polsiere	12# - Using wrist restraints	0.86	<.0001	very good
13# - Attraverso l'uso di dispositivi fai-da-te (lenzuola/fasce/bende)	13# - Through the use of DIY devices (sheets/bandages/bandages)	0.81	<.0001	very good

14# - Attraverso la sedazione farmacologica	14# - Using pharmacological sedation	0.69	<.0001	good
15# - Essere ostacolata da colleghi/e quando voglio rimuovere la contenzione applicata	15# - I face resistance from colleagues when I want to remove the restraints applied to a patient	0.64	<.0001	good
16# - Essere in disaccordo con i colleghi/e e altri membri dello staff in tema di contenzione	16# - I find myself in disagreement with colleagues and other staff members regarding restraint	0.62	<.0001	good
17# - Mantenere la contenzione ad un paziente quando è stata applicata da un altro collega	17# - I have to maintain restraint on a patient when it has been applied by another colleague	0.67	<.0001	good
18# - Mi sento a disagio nell'applicare la contenzione ad un paziente anche se per garantirne la sicurezza	18# - I would feel uncomfortable applying restraints to a patient even if it is to ensure their safety	0.74	<.0001	good
19# - In terapia intensiva non è possibile evitare completamente la contenzione	19# - In intensive care it is not possible to completely avoid restraint	0.79	<.0001	good
20# - La decisione di contenere un paziente deve essere condivisa con il resto dell'equipe	20# - The decision to restrain a patient should be shared with the rest of the team	0.92	<.0001	very good
21# - La famiglia del paziente deve essere informata sui motivi che hanno portato alla contenzione	21# - The patient's family must be informed of the reasons that led to the restraint	0.92	<.0001	very good
22# - Gli infermieri che lavorano in terapia intensiva non dovrebbero mai applicare la contenzione	22# - Nurses working in intensive care should never apply restraints	0.79	<.0001	good
23# - La famiglia del paziente non ha il diritto di opporsi alla contenzione quando è applicata per garantire la sicurezza dello stesso	23# - The patient's family does not have the right to oppose restraint when it is applied to ensure the patient's safety	0.70	<.0001	good
24# - La famiglia può essere formata a stare accanto al paziente al fine di evitare la contenzione in terapia intensiva	24# - The family can be trained to stay close to the patient in order to avoid restraint in intensive care	0.84	<.0001	very good
25# - La contenzione in terapia intensiva può essere applicata quando non si riesce a garantire l'osservazione costante di un paziente agitato	25# - Restraint in intensive care may be applied when constant observation of an agitated patient cannot be guaranteed	0.74	<.0001	good
26# - La contenzione si può applicare quando il paziente è confuso o agitato	26# - Restraint can be applied when the patient is confused or agitated	0.64	<.0001	good

27# - La contenzione se applicata deve essere documentata in cartella indicandone la motivazione, l'orario di inizio e di fine e la modalità con il distretto corporeo interessato ad ogni cambio di servizio	27# - If restraint is applied, it must be documented in the chart indicating the reason, the start and end time and the method with the body area involved at each change of service	0.86	<.0001	very good
28# - In terapia intensiva, la contenzione garantisce la sicurezza del paziente	28# - In intensive care, restraint ensures patient safety	0.65	<.0001	good
29# - La contenzione può essere causa di gravi complicanze	29# - Restraint can cause serious complications	1.00	<.0001	very good
30# - La contenzione può essere causa di morte	30# - Restraint can be a cause of death	0.95	<.0001	very good
31# - In paziente confuso ed agitato non esistono alternative valide alla contenzione	31# - In a confused and agitated patient there are no valid alternatives to restraint	0.63	<.0001	good
32# - L'infermiere che applica la contenzione può essere perseguito per legge	32# - The nurse who applies restraint can be prosecuted by law	0.81	<.0001	very good

*Altman DG (1991) Practical statistics for medical research. Chapman & Hall: London
