# Supplementary material for: Monitoring Knowledge, Attitudes, and Practices on Restraint Use in Adult and Pediatric Intensive Care Units: The Multicenter Development and Validation of the CON-Ti-IT Questionnaire
Source: Nurs Rep. 2025 Dec 25;16(1):10. doi: 10.3390/nursrep16010010 (PMC12844329; doi:10.3390/nursrep16010010)
Supplement: Supplementary file 1 [file nursrep-16-00010-s001.zip › Supplementary File_S2.pdf]

Supplementary File S2. CON-Ti-IT (English version)

Dear participant,

the following statements explore the practice, knowledge, and attitudes of nurses working in the ICU regarding restraint. Consider each of the following statements in relation to your personal experience and select the most appropriate response according to the directions posed in the text.

QUESTIONNAIRE

STATEMENTS	VALUES				
By marking with an X in the desired box, assign a value from "Never" (1) to "Always" (5) to the following statements:<br><br>In my work in intensive care unit, I happen to:	Never<br><br>1	2	3	4	Always<br><br>5
1) restrain a patient upon as they wake up from general anesthesia					
2) restrain a patient when they are in an altered mental state					
3) restrain a patient when in a state of psychomotor agitation					
4) restrain a patient to prevent a fall from the bed					
5) restrain a patient to prevent a fall from the chair or armchair					
6) restrain a patient to prevent the removal of a life-saving device (e.g., endotracheal tube)					
7) restrain a patient to prevent the removal of devices (e.g., nasogastric tube, bladder catheter)					
8) restrain a patient when constant observation cannot be guaranteed (e.g., during meetings/handovers)					
9) restrain a patient when the workload in intensive care is high (e.g., ongoing emergency)					
10) restrain a patient in case of inadequate nurse/patient ratio for the complexity (e.g., lack of staff)					
11) restrain a patient when the team deems it necessary					
12) restrain a patient using wrist restraints					
13) restrain a patient through the use of DIY devices (sheets/bandages/bandages)					
14) restrain a patient using pharmacological sedation					
15) face resistance from colleagues when I want to remove the restraints applied to a patient					
16) find myself in disagreement with colleagues and other staff members regarding restraint					
17) maintain restraint on a patient when it has been applied by another colleague					
Now, by marking with an X in the desired box, assign a value from "I strongly agree" (1) to "I strongly disagree" (5) to the following statements:	I strongly agree<br>1	2	3	4	I strongly diagree<br>5
18) I would feel uncomfortable applying restraints to a patient even if it is to ensure their safety					
19) In intensive care it is not possible to completely avoid restraint					
20) The decision to restrain a patient should be shared with the rest of the team					
21) The patient's family must be informed of the reasons that led to the restraint					
22) Nurses working in intensive care should never apply restraints					
23) The patient's family does not have the right to oppose restraint when it is applied to ensure the patient's safety					

Now, by marking with an X in the desired box, assign a value between "YES, NO, I DON'T KNOW" to the following statements:	YES	NO	I DON'T KNOW		
24) The family can be trained to stay close to the patient in order to avoid restraint in intensive care					
25) Restraint in intensive care may be applied when constant observation of an agitated patient cannot be guaranteed					
26) Restraint can be applied when the patient is confused or agitated					
27) If restraint is applied, it must be documented in the chart indicating the reason, the start and end time and the method with the body area involved at each change of service					
28) In intensive care, restraint ensures patient safety					
29) Restraint can cause serious complications					
30) Restraint can be a cause of death					
31) In a confused and agitated patient there are no valid alternatives to restraint					
32) The nurse who applies restraint can be prosecuted by law					
