# Supplementary material for: Monitoring Knowledge, Attitudes, and Practices on Restraint Use in Adult and Pediatric Intensive Care Units: The Multicenter Development and Validation of the CON-Ti-IT Questionnaire
Source: Nurs Rep. 2025 Dec 25;16(1):10. doi: 10.3390/nursrep16010010 (PMC12844329; doi:10.3390/nursrep16010010)
Supplement: Supplementary file 1 [file nursrep-16-00010-s001.zip › Supplementary File_S3.pdf]

Supplementary File S3. CON-Ti-IT (Italian version)

Gentile partecipante,

di seguito sono riportate alcune affermazioni che vanno a esplorare la pratica, la conoscenza e gli atteggiamenti degli infermieri impegnati in terapia intensiva in merito alla contenzione. Considera ognuna delle seguenti affermazioni in relazione alla tua esperienza personale e seleziona la risposta più idonea secondo le indicazioni che ti verranno poste nel testo.

QUESTIONARIO

AFFERMAZIONI	VALORI				
Segnando con una X nella casella desiderata, assegna un valore da "Mai"(1) a "Sempre"(5) alle seguenti affermazioni:<br><br>"Nel mio lavoro in terapia intensiva mi capita di….":	Mai<br><br>1	2	3	4	Sempre<br><br>5
1) contenere un paziente al suo risveglio da un'anestesia totale					
2) contenere un paziente quando è in uno stato confusionale					
3) contenere un paziente quando è in uno stato di agitazione psicomotoria					
4) contenere un paziente per prevenire una caduta dal letto					
5) contenere un paziente per prevenire una caduta dalla sedia o dalla poltrona					
6) contenere un paziente per evitare che si rimuova un device salvavita (es. tubo endotracheale)					
7) contenere un paziente per evitare che si rimuova device (es. sondino naso gastrico, catetere vescicale)					
8) contenere un paziente quando non è possibile garantire un'osservazione costante (ad es. durante riunioni/consegne)					
9) contenere un paziente quando il carico di lavoro in terapia intensiva risulta elevato (es. emergenza in corso)					
10) contenere un paziente in caso di rapporto numerico infermieri/pazienti inadeguato alla complessità (es. mancanza di personale)					
11) contenere un paziente quando l'equipe lo ritiene necessario					
12) contenere un paziente attraverso l'uso di polsiere					
13) contenere un paziente attraverso l'uso di dispositivi fai-da-te (lenzuola/fasce/bende)					
14) contenere un paziente attraverso la sedazione farmacologica					
15) essere ostacolato/a da colleghi/e quando voglio rimuovere la contenzione applicata					
16) essere in disaccordo con i colleghi/e e altri membri dello staff in tema di contenzione					
17) mantenere la contenzione ad un paziente quando è stata applicata da un altro collega					
Ora, segnando con una X nella casella desiderata, assegna un valore da "sono pienamente d'accordo" (1) a "non sono d'accordo" (5) alle seguenti affermazioni :	Sono pienamente d'accordo<br>1	2	3	4	Non sono d'accordo<br><br>5
18) Mi sento a disagio nell'applicare la contenzione ad un paziente anche se per garantirne la sicurezza					
19) In terapia intensiva non è possibile evitare completamente la contenzione					
20) La decisione di contenere un paziente deve essere condivisa con il resto dell'equipe					
21) La famiglia del paziente deve essere informata sui motivi che hanno portato alla contenzione					

22) Gli infermieri che lavorano in terapia intensiva non dovrebbero mai applicare la contenzione					
23) La famiglia del paziente non ha il diritto di opporsi alla contenzione quando è applicata per garantire la sicurezza dello stesso					
Ora, segnando con una X nella casella desiderata, assegna un valore tra "SI, NO, NON SO" alle seguenti affermazioni	SI	NO	NON SO		
24) La famiglia può essere formata a stare accanto al paziente al fine di evitare la contenzione in terapia intensiva					
25) La contenzione in terapia intensiva può essere applicata quando non si riesce a garantire l'osservazione costante di un paziente agitato					
26) La contenzione si può applicare quando il paziente è confuso o agitato					
27) La contenzione se applicata deve essere documentata in cartella indicandone la motivazione, l'orario di inizio e di fine e la modalità con il distretto corporeo interessato ad ogni cambio di servizio.					
28) In terapia intensiva, la contenzione garantisce la sicurezza del paziente					
29) La contenzione può essere causa di gravi complicanze					
30) La contenzione può essere causa di morte					
31) In paziente confuso ed agitato non esistono alternative valide alla contenzione					
32) L'infermiere che applica la contenzione può essere perseguito per legge					
